# Fostering critical thinking skills: a strategy for enhancing evidence based wellness care

**DOI:** 10.1186/1746-1340-13-19

**Published:** 2005-09-08

**Authors:** Jennifer R Jamison

**Affiliations:** 1 School of Chiropractic, Murdoch University, South Street, Perth, Western Australia, 6849, Australia

**Keywords:** Chiropractic, critical thinking skills, wellness

## Abstract

Chiropractic has traditionally regarded itself a wellness profession. As wellness care is postulated to play a central role in the future growth of chiropractic, the development of a wellness ethos acceptable within conventional health care is desirable.

This paper describes a unit which prepares chiropractic students for the role of "wellness coaches". Emphasis is placed on providing students with exercises in critical thinking in an effort to prepare them for the challenge of interfacing with an increasingly evidence based health care system.

**Methods:**

This case study describes how health may be promoted and disease prevented through development of personalized wellness programs. As critical thinking is essential to the provision of evidence based wellness care, diverse learning opportunities for developing and refining critical thinking skills have been created. Three of the learning opportunities are an intrinsic component of the subject and, taken together, contributed over 50% of the final grade of the unit. They include a literature review, developing a client wellness contract and peer evaluation. In addition to these 3 compulsory exercises, students were also given an opportunity to develop their critical appraisal skills by undertaking voluntary self- and unit evaluation. Several opportunities for informal self-appraisal were offered in a structured self-study guide, while unit appraisal was undertaken by means of a questionnaire and group discussion at which the Head of School was present.

**Results:**

Formal assessment showed all students capable of preparing a wellness program consistent with current thinking in contemporary health care. The small group of students who appraised the unit seemed to value the diversity of learning experiences provided. Opportunities for voluntary unit and self-appraisal were used to varying degrees.

Unit evaluation provided useful feedback that led to substantial changes in unit structure.

**Conclusion:**

Students have demonstrated themselves capable of applying critical thinking in construction of evidence based wellness programs. With respect to unit design, selective use of learning opportunities highlighted the desirability of using obligatory learning opportunities to ensure exposure to core constructs while student feedback was found to provide useful information for enriching unit review.

It is hoped inclusion of critical thinking learning opportunities in the undergraduate chiropractic curriculum will contribute to the development of an evidence based ethos in chiropractic care.

## Background

Health care has long been regarded as an art and a science. In contemporary conventional health care the 'science' dimension has increasingly come to dominate the 'art' of health care. At the undergraduate level this has been expressed as enhanced emphasis in the training of future physicians on searching and critically evaluating the available literature utilizing electronic and other databases [1]. At the level of the health care system allopathic disciplines are encouraging critical and empirical evaluation of alternative medical techniques [2,3]. Evidence based medicine {EBM} has become the new health care mantra and is largely pursued through critical evaluation of individual research studies, systematic reviews of studies in a particular area or practice, evidence-based practice guidelines outlining standards for the profession, and evidence-based systems of care focusing on implementation [4]. In each of these pursuits critical thinking emerges as a requisite skill.

Despite chiropractic's philosophy of vitalism contrasting sharply with the "mechanistic" foundations of orthodox medicine, there are some in the chiropractic profession who welcome this development. Not only may the development of evidence-based guidelines in chiropractic practice insulate against malpractice lawsuits, they may also improve relations between chiropractic and the health care system and better enable the chiropractic profession achieve is foremost goal of serving as a portal of entry into the health care system with chiropractors functioning as primary contact practitioners.

In addition to chiropractic functioning at the community-health care system interface [5], the chiropractic profession considers itself a provider of wellness care and this is subsumed under the mantel of maintenance care [6]. "Maintenance" or "wellness" care involves regular, ongoing visits that is not correlated directly to symptomatology. However George B. McClelland, DC, Chairman ACA Board of Governors has stated "Philosophically the idea of regular spinal manipulative therapy opposes the concept of wellness" [7]. Furthermore it has been suggested that: "...the proposition of chiropractic as a "wellness profession" is not defensible." [8]. Conventional health care would concur given that there are those in the chiropractic profession whose practice of wellness care is limited to correcting subluxations. While the notion that mechanical and functional disorders of the spine, expressed as subluxations, can degrade health and correction of spinal disorders by adjustments may restore health is fundamental to chiropractic thinking, there is no scientifically acceptable data to support this belief. Furthermore, wellness care calls for a holistic approach and the desirability for the chiropractic profession to explore a more comprehensive approach to wellness care is apparent given the Institute of Alternative Futures report *Future of Chiropractic Revisited: 2005 to 2010*, which suggested possible growth scenarios for chiropractic were as "wellness coaches" or as "healthy life doctors" with a wellness mindset.

If chiropractic is to evolve as a wellness profession in an increasingly evidence based health care system, it would seem necessary that it critically appraise its current wellness practices and adopt a schema in which its practitioners serve as motivators and educators. One initiative which may contribute to this end is to include in undergraduate education units which encourage critical thinking in the context of health promotion and disease prevention. Murdoch university provides their third year chiropractic students with just such a learning opportunity.

Critical thinking skills are thoughtfully being incorporated into the curriculum of nursing [9,10] and medical programs [11], at both under- and post graduate levels [12-14].

Critical thinking is regarded as purposeful, self-regulatory judgment. In addition to evaluating whether arguments are strong, weak or relevant, critical thinking involves inferring degrees of truth from given data; recognizing unstated assumptions underlying assertions; deducing whether conclusions necessarily follow from given statements and interpreting and weighing evidence to decide if generalizations are warranted [15]. It is commonly accepted that critical thinking can be taught. Diverse learning opportunities have been shown to facilitate the development and acquisition of this skill ranging from concept mapping [10], through critical questioning workshops [11] and systematic literature reviews [13] to problem based learning [14]. Problem based learning programs create scenarios in which prior knowledge is activated in a meaningful context thereby encouraging elaboration and organization of knowledge [16]. Students in problem based curricula demonstrate an enhanced ability to apply science based concepts to their explanations [17]. While problem based learning appears to be particularly useful for refining reasoning skills, integration of critical thinking in all areas of learning has been found a useful strategy for fostering this ability [18].

This paper describes how a preclinical unit has been structured to include diverse learning opportunities for applying critical thinking skills in the context of wellness. It illustrates how students can be given opportunities to practice critical thinking as a prelude to practicing evidence based health care.

## Case Presentation

### Unit Design

Health Promotion and Nutritional Management is a subject taught in the third year of a 5 year chiropractic program at Murdoch University. The broad aims of this unit are to:

1. Provide the student with a strategy for implementing personal wellness programs in clinical practice.

2. Enable the student to critically explore the contribution of lifestyle interventions, including the use of nutrients in therapeutic doses, in health promotion, disease prevention and management.

3. Alert the student to the early signs and symptoms suggestive of some lifestyle modifiable diseases prevalent in primary practice.

The learning objectives are to:

• Enhance wellness through recruitment of wellness triggers; identification and reduction of lifestyle risk factors; promotion of fitness; and provide early diagnosis and management, using lifestyle interventions and nutritional therapy, of selected diseases prevalent in primary practice.

• Empower patients to take increased personal responsible for their health care through formulation of wellness contracts by performing a personal health status appraisal; screening patients to ascertain their risk of prevalent diseases; negotiating health goals through examination of patient's perceived and professionally assessed health needs; determining potential barriers, including cultural, socio-economic factors, to implementation of health promotion and disease prevention strategies; negotiating a health promotion and disease prevention plan; implementing a personalized health management program; monitoring patient progress and modify the health contract, as required.

• Analyze the patient's preferred interaction style and adapt ones mode of clinical care as required.

• Critically appraise relevant literature and apply evidence-based problem solving to promote wellness.

• Implement a self-care wellness program.

The unit provides a classroom learning experience which runs for 6 weeks, and a structured self-learning guide, complemented by WebCT, a computer based learning platform, which runs for 13 weeks of the semester. The unit has been designed to enhance active and encourage independent learning and provides 5 distinct opportunities for developing and refining critical thinking skills. The 5 critical thinking opportunities provided ranged from client health assessment, peer evaluation and literature review, which together contribute almost 60% of the final grade, to voluntary self-assessment and finally unit evaluation.

#### 1 – Self-Assessment

**Figure 1:**
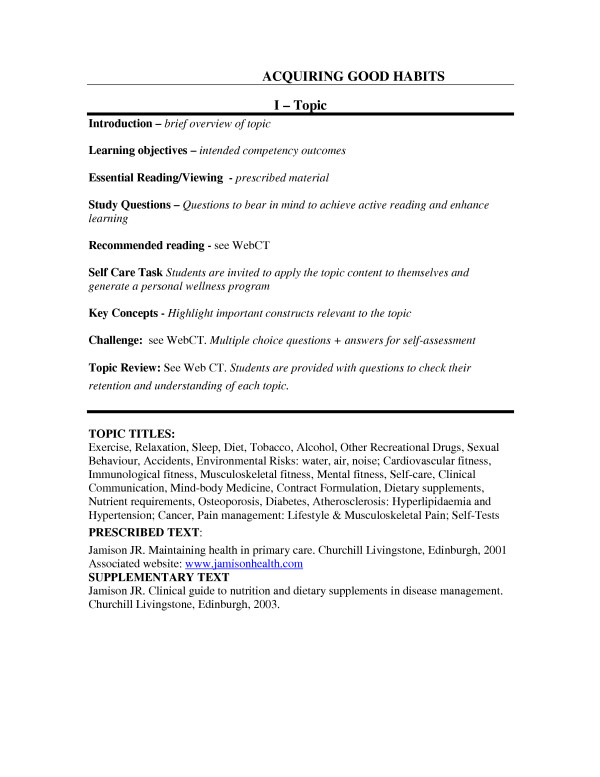
Acquiring good habits.

The self-assessment learning experiences are embedded in the structured self-study learning guide. The learning guide has been structured to provide students with a opportunity to undertake continuous formative self-assessment. Figure 1 shows the template used in the structured self-directed learning guide and depicts the guideposts to the self-assessment critical appraisal opportunities provided by the challenge and review questions and self-care tasks. The factual content of the unit is covered in 25 discrete topics each of which contains a unique learning template. For each topic the student is provided with self-assessment opportunities to:

• Critically review their learning by completing challenge and review questions based on the content of that topic. The student has the opportunity to monitor their grasp and recall of factual information.

• Apply the information provided in that topic to their lifestyle and formulate a personal wellness program. The student is given the opportunity to preview construction of a wellness program in a non-threatening environment and simultaneously embrace a self-care system based on a lifetime of health choices.

#### 2 – A Client Wellness Program

**Figure 2:**
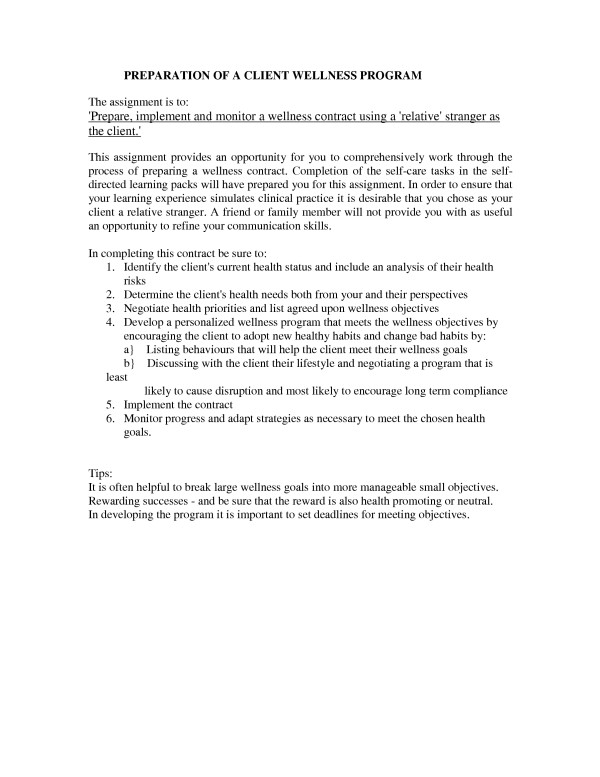
Preparation of client wellness program.

Students who chose to prepare a personal wellness program are particularly well prepared when required to formulate the formal client wellness program. Formulating a wellness program for a client passes through a number of critical thinking steps. Students are required to undertake critical appraisal of a client's lifestyle with respect to their good and bad habits and, given their family history, ascertain the client's health risk. They are then required to identify health needs and, in negotiation with the client, develop a list of wellness goals. The next steps are to make the client aware of diverse strategies for achieving these goals, help them select and then implement those strategies appropriate to their lifestyle. The student is then required to monitor the client's wellness program and adapt the program as needed to meet ongoing client successes, failures and changing needs. See Figure 2.

#### 3 – Peer Evaluation

**Figure 3:**
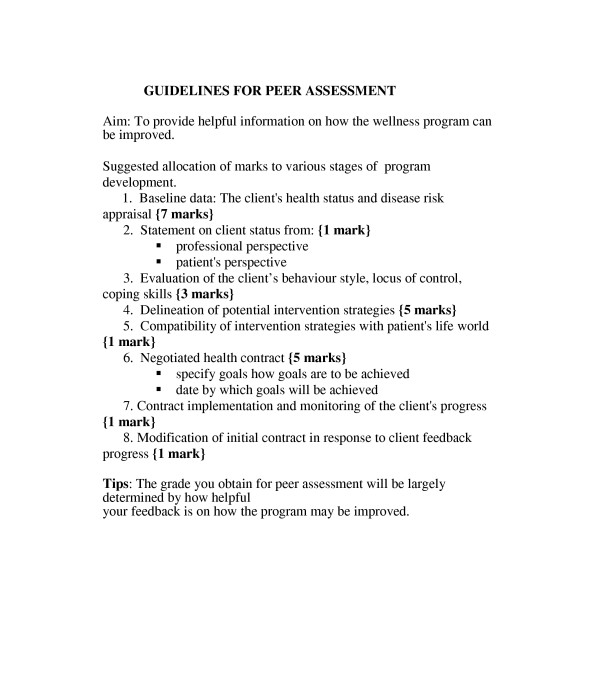
Guidelines for peer assessment.

The peer evaluation task is closely linked to the wellness program. Students are asked to appraise the wellness contract prepared by another student. They are encouraged to analyze all aspects of the program with a view to making useful suggestions on how the program may be improved. See figure 3. Marks are scored for constructive criticisms that provide feedback which enhances the learning of the program originator and potentially improves the wellness outlook of the client.

#### 4 – Literature Appraisal

**Figure 4:**
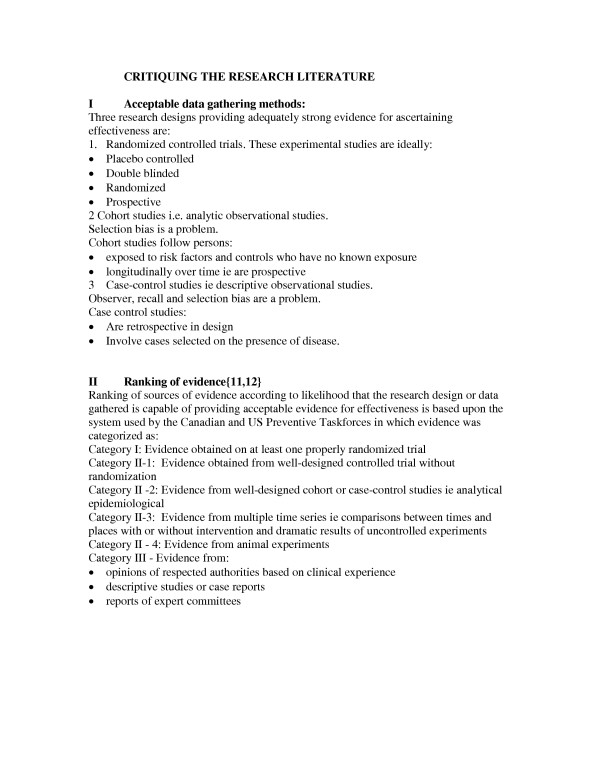
Critiquing the research literature.

The ability to assess the scientific validity of information is increasingly recognized as an essential competence in a profession which is increasingly embracing the notion of evidence based practice. It is therefore imperative that students are given opportunities to critically evaluate the literature. For this exercise students are required to rank evidence according to the system developed by the Canadian Task Force and the US Preventive Services Task Force [19,20]. The guidelines for the nutritional literature review included as part of the students' formal in this unit can be found in Figure 4[21].

Along with the client wellness program and its critique, the students' literature review contributes over half of the total grade for the unit.

#### 5 – Unit Appraisal

**Figure 5:**
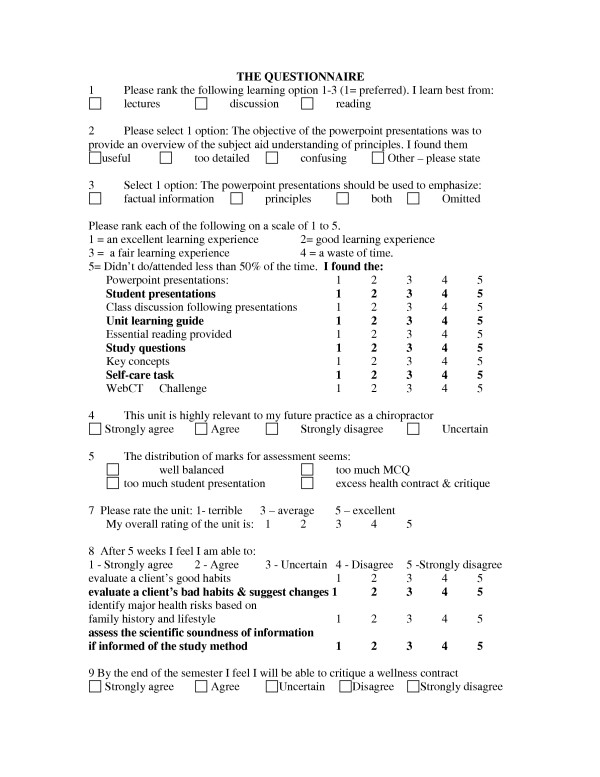
The questionnaire.

In contrast to peer-, client- and literature assessment, students are given an optional opportunity to critically appraise the unit. Unit appraisal takes two forms. An informal questionnaire survey of student opinion initiated by the lecturer, see Figure 5, and a formal group discussion. All students are invited to participate in the group discussion which forms part of the formal School's assessment of the unit. The Head of School is present for and leads these discussions.

## Results

Summative student assessment found students could competently prepare a client wellness program. Analysis of client wellness programs submitted for formal assessment confirmed that students had mastered the skills required to achieve this objective. All students demonstrated the ability to appraise their client's lifestyle, prepare and monitor a wellness program Most students were demonstrably competent to ascertain their client's individual disease risk or health hazard as based on a family history and lifestyle. All but 2 students commented on the preferred behaviour style of their client and took this into consideration when formulating their wellness program. A few students took their own preferred behaviour style into consideration and analysed how this may be modified to best suit the client.

In contrast to their success at developing a wellness program, formal assessment of the peer appraisal assignment suggested they found critiquing a wellness program more demanding than constructing one. While all students provided satisfactory comment on the structure and content of an others wellness program, some students faltered when required to provide useful information for refining the initial program.

Formal assessment of the students' critical appraisal of the literature found all students capable of searching the literature and extracting relevant papers. Furthermore, most students were able to compare and discuss conflicting research reports and many showed themselves capable of commenting on potential biases resulting from flaws in research design. However, few categorized the level of evidence provided according to the schema proposed by the Canadian and US Preventive Taskforces.

In contrast to the above compulsory critical thinking opportunities, few students availed themselves of the opportunities offered for unit assessment. The unit survey provided insight into the students' appraisal of the unit as a whole as well as specifically provided feedback on their evaluation of various critical thinking opportunities. Of a class of some 60 students, a total of 22 completed the survey. Consistent with the ethos of independent learning, attendance is optional except when students are required to present their critique of the nutrition literature. The unit survey was completed by 17 students who voluntarily attended lectures and by a further 5 students who were required to do their class presentation on the day of the survey.

**Figure 6:**
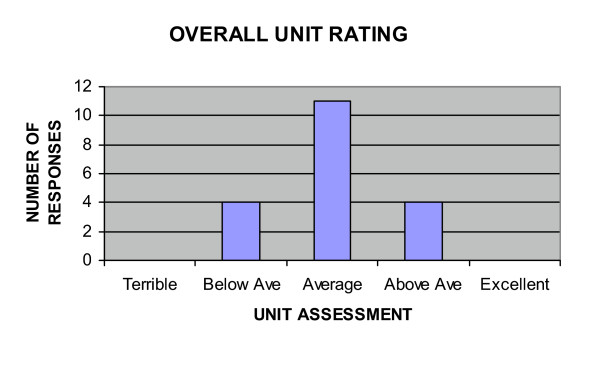
Overall unit rating.

**Figure 7:**
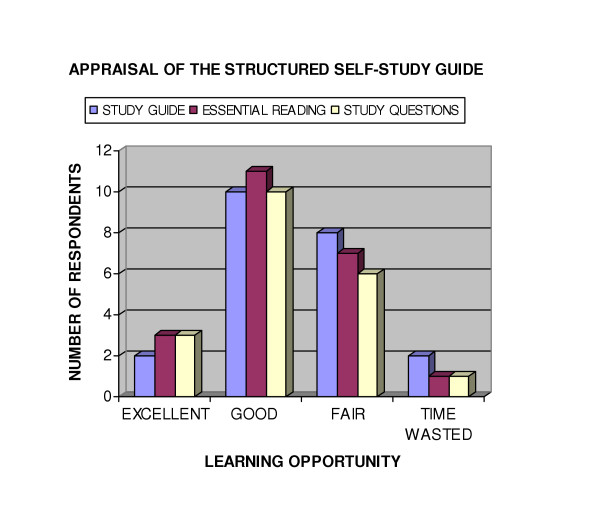
Appraisal of the Structured Self-study guide.

Half the students participating selected lectures as their most preferred learning style, a finding verified when ranked preferences were analyzed on a Likert type scale. Figure 6 describes the overall unit rating. Eighteen students regarded the unit as highly relevant to their future practice as a chiropractor, 3 were uncertain and 1 felt it was irrelevant. The students' self-assessment of their critical reading/learning opportunity is reported in Figure 7 which provides an overview of the perceived usefulness of the study guide, the essential reading and study questions. Linking study questions with the unit's content provided an opportunity for active learning and critical interpretation of new information. It also provided an opportunity for self-assessment. Two students indicated they had not attempted any of the study questions.

**Figure 8:**
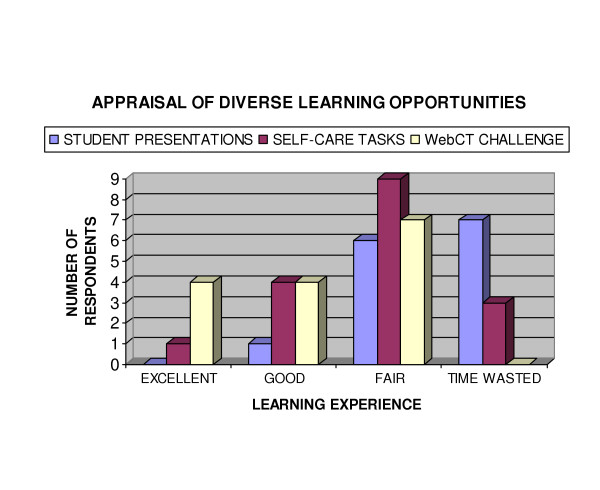
Appraisal of Diverse Learning Opportunities.

A Likert type scale was used to ascertain which of the learning experiences students perceived as most valuable. Students who indicated they hadn't performed or who had attended less than half of the sessions offered for a particular activity were deemed unqualified to comment and excluded from analysis of that activity. A score of 5 per student was allocated to each activity rated as an excellent learning experience, 4 was allocated for an activity rated as good, 3 for a fair learning experience and 2 per student for activities rated as a waste of time. The score derived was then divided by the number of respondents to that item and the final score was used to rank learning experiences. On this arbitrary scale the most valued learning experiences, WebCT challenge and study questions, each achieved a total of 3.8; the least appreciated, student presentation, a value of 2.57. Figure 8 shows how students appraised the popular WebCT challenge compared to the self-care and student presentation learning experiences. The WebCT challenge provided students with a formative self-assessment opportunity to evaluate the acquisition of factual knowledge which would be later tested in formal summative examination of the unit. Despite this imperative, 7 students had not used the WebCT challenge, similarly 7 had not implemented any self-care tasks.

**Figure 9:**
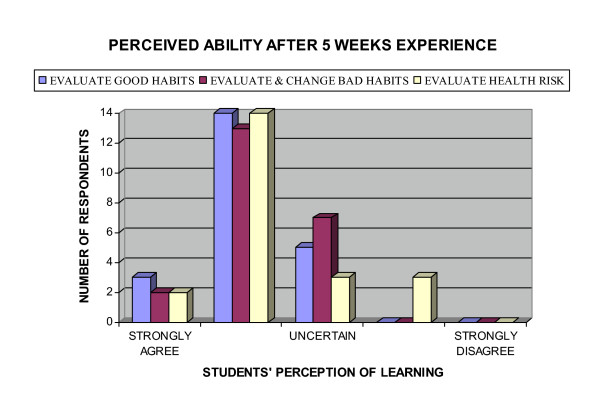
Perceived ability after 5 weeks: Students perception of learning.

This trend extended to student presentation. Five {5} respondents indicated they had attended less than half the possible student presentations. Student presentations emerged, both in the questionnaire and in small group evaluation of the unit, to be regarded as 'a waste of time'. Clarification identified that although students found the literature search and data analysis to be useful, the classroom format was regarded as 'boring' and too time consuming. This perspective was confirmed by the group of 6 students who attended the formal unit assessment conducted by the Head of School. Despite the negative classroom learning experience, the students attending the formal unit evaluation indicated they regarded the ability to critically analyze the literature an important component of their training. Furthermore, as shown in Figure 9, two out of 3 respondents felt they had the analytical skills to assess the scientific validity of information if they were provided with details of the research methods used, a perception was verified on formal assessment.

**Figure 10:**
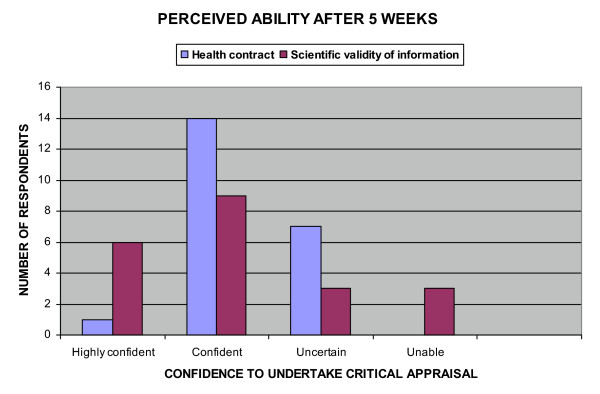
Perceived ability after 5 weeks: Confidence to undertake critical appraisal.

Based on the learning they had experienced during the first 5 weeks of the semester, students were asked whether they believed themselves capable of preparing a client wellness contract. Figure 9 shows the majority of students judged themselves capable of evaluating a client's good habits, determining and changing a client's bad habits and assessing and performing a non-invasive health hazard appraisal. Formal assessment confirmed their optimism. In contrast the confidence of respondents with regard to their ability to undertake peer evaluation, see Figure 10, was not confirmed on formal assessment.

## Discussion

While it is unclear whether the correction of subluxations makes a unique contribution to wellness, it is apparent that care beyond an adjustment is required if chiropractors are to take the role of 'wellness coaches' or "healthy life doctors" in conventional health care. Wellness is a growth industry and the scientific basis of many wellness practices is uncertain. Critical thinking is fundamental to and regarded an important educational objective in the preparation of health professionals as evidence based carers [22]. Problem based learning scenarios have been found to be conducive to developing critical thinking skills in the classroom [14-18] and on the internet [23]. This paper described how by combining classroom interaction with paper based and internet self-study opportunities various learning opportunities have been created to enhance critical thinking in a wellness context.

Upon completion of the unit, formal assessment found students capable of formulating and administering a client wellness program, undertaking peer review and critically appraising the literature. These findings were largely consistent with the perceptions of the small group of students who chose to evaluate the unit. While any extrapolation of the results of the unit evaluation to the whole class is precluded due to the small size of the participating group, the results of this exercise did provide useful information for future planning. Marked discrepancies emerged with respect to the preferred learning opportunities of different students in the respondent group. Given that the majority of students completing the unit assessment were voluntarily attending a classroom learning experience, it was perhaps not surprising that overall they indicated a clear preference for lecture based learning. It seems not unreasonable to surmise that at least some of their colleagues, who chose to omit classroom learning, preferred a more independent scenario. When structuring a unit it may therefore be prudent to consider providing diverse learning scenarios for acquiring similar knowledge, skills and attitudes to cater for the learning needs of different individuals. Another red flag which emerged from this study is the necessity to incorporate compulsory learning opportunities. Although WebCT and self-study questions were the learning opportunities most favored by the majority of respondents, there were those who had not utilized these learning measures. While students with different learning styles may be expected to avail themselves of different learning opportunities, it should be noted that students were aware that these self-assessment learning experiences covered content in a format similar to the proposed end of semester examination. As some students, despite this incentive chose to omit these learning experiences the need for compulsory completion of selected learning task seems advantageous. In unit planning, it would certainly seem desirable to ensure that knowledge and skills considered fundamental to chiropractic practice are included in diverse obligatory tasks.

Consistent with the ethos of student centred learning, student unit evaluation provides useful feedback for future planning. In this instance, unit modifications in response to criticisms leveled at the format of the student presentations promises to enrich the unit for future students. While retaining the central theme of demonstrating proficiency in critically appraising the literature, the delivery mode will be modified from student presentation to student debate. For example, instead of being asked to discuss the scientific basis for the use of Echinacea, the challenge will be for 2 teams to use scientifically justifiable arguments for and against the statement "Echinacea can be used to prevent the common cold".

## Conclusion

This paper described diverse learning experiences designed to enhance critical thinking skills in the context of wellness. By using various modalities in diverse problem solving formats the classroom, internet and a study guide have been combined to create independent, structured self-learning situations. Results of summative student assessment showed students capable of developing a personalized client wellness program consistent with current thinking in conventional health care. By providing a diversity of critical thinking learning opportunities, the more fundamental of which are compulsory, it is hoped that this unit will contribute to the graduation of chiropractors better prepared to interface as 'wellness coaches' or 'healthy life doctors' within an evidence based health care system.
